# *GLIS3* and Thyroid: A Pleiotropic Candidate Gene for Congenital Hypothyroidism

**DOI:** 10.3389/fendo.2018.00730

**Published:** 2018-11-29

**Authors:** Giuditta Rurale, Luca Persani, Federica Marelli

**Affiliations:** ^1^Department of Clinical Sciences and Community Health, University of Milan, Milan, Italy; ^2^Division of Endocrine and Metabolic Diseases & Laboratory of Endocrine and Metabolic Research, IRCCS Istituto Auxologico Italiano, Milan, Italy

**Keywords:** *GLIS3*, sonic hedgehog, thyroid, congenital hypothyroidism, zebrafish

## Abstract

Variations in the transcription factor Gli-similar 3 (*GLIS3*) gene have been associated to variable congenital endocrine defects, including both morphogenetic and functional thyroid alterations. Evidence from Glis3 knockout mice indicates a relevant role for *GLIS3* in thyroid hormone biosynthesis and postnatal thyroid gland growth, with a mechanism of action downstream of the TSH/TSHR interaction. However, the pathophysiological role of this transcription factor during the embryonic thyroid development remains unexplored. In this manuscript, we will provide an overview of the current knowledge on *GLIS3* function during development. As a perspective, we will present preliminary evidence in the zebrafish model in support of a potential role for this pleiotropic transcription factor in the early stages of thyroid gland development.

## *GLIS3* in human pathophysiology

The transcription factor Gli-similar 3 (*GLIS3*) belongs to the family of the Krüppel-like zinc finger transcription factor (1) and it is involved in several cellular processes, including proliferation, apoptosis, differentiation and development (2).

The human *GLIS3* gene is located on chromosome 9p24.2 and encodes a full-length protein of 90 kD in size. *GLIS3* in expressed in a tissue-specific manner, with highest levels in kidney, thyroid gland, endocrine pancreas, thymus, testis, and uterus. Lower levels of expression were also described in brain, lung, ovary, and liver (1, 3).

Several alternate transcripts of *GLIS3* have been reported so far in humans. Nine out of nineteen isoforms are processed into proteins with unknown functions. The larger transcript (7.5 kb) is predominantly expressed in pancreas, thyroid, and kidney, whereas the smaller isoforms (0.8–2 kb) are mainly localized in heart, kidney, liver and skeletal muscle (4). Despite the distinct pattern of expression, so far there is no evidence of variable pathophysiological functions for these different transcripts.

The *GLIS3* protein consists of a centrally located zinc finger domain (ZFD) (five tandems Cys_2_-His_2_ zinc finger motifs), a C-terminal transactivation domain (TAD) and a relatively large N-terminus that is important in the regulation of *GLIS3* stability and its transcriptional activity (1, 5).

The ZFD dictates *GLIS3* cytosolic or nuclear localization and is responsible for the recognition of specific DNA responsive elements, the GlisBS (Glis-binding sites) are located within the regulatory regions of target genes (1).

Once bound to the DNA, *GLIS3* can repress or enhance the expression of target genes (1, 6).

Furthermore, *GLIS3* stability is regulated by the binding with the “Suppressor of Fused” (SUFU), a negative regulator of Hedgehog (SHH) signaling, via a YGH motif localized into the N-terminal domain (7). SUFU not only protects Glis3 from proteasomal degradation stabilizing Glis3 protein (8), but also modulates Glis3-mediated transactivation of the promoter of target genes (9).

Based on the current knowledge, *GLIS3* represents a new candidate gene for congenital hypothyroidism (CH) but its role in thyroid development and function remains largely unexplored.

Homozygous and compound heterozygous mutations in the *GLIS3* gene have been associated with a rare syndrome, called NDH, characterized by neonatal diabetes (ND) and CH.

NDH patients frequently present a polycystic kidney disease, and are variably affected by additional abnormalities, such as developmental delay, hepatic fibrosis, congenital glaucoma, and facial dimorphism (1, 3, 10, 11).

The *GLIS3* alterations identified in NDH patients included missense substitutions, frameshift mutations resulting in premature termination, deletions encompassing exons 5–9, 3–4, 9–11, 10–11 and larger deletions covering regions >100 kb that include exons 1–2, 1–4, or 5–9 (8).

A Chinese group recently screened *GLIS3* gene in 592 CH patients and 600 controls by Next Generation Sequencing (NGS) and Multiplex Ligation-dependent Probe Amplification (MLPA) (12). They found two different variations (one deletion at chromosome 9p24.3p23, including the *GLIS3* gene, and one missense variant (p.R720Q) in two CH patients with gland-*in-situ* (frequency of *GLIS3* variations in Chinese CH: 0.3%) but not in controls. Both variations were in the heterozygous state (12).

We have recently identified rare heterozygous *GLIS3* missense variants by NGS in a large cohort (18/177) of Caucasian patients with isolated CH (13). Clinically, half of the affected cases presented variable thyroid dysgenesis (athyreosis, thyroid hypoplasia, and ectopy), whereas the remaining part have *in situ* thyroid gland (13). Interestingly, these *GLIS3* variants are associated in all 18 cases with other rare variations in genes involved in thyroid pathology, supporting a frequent oligogenic origin of CH.

**Figure 1:**
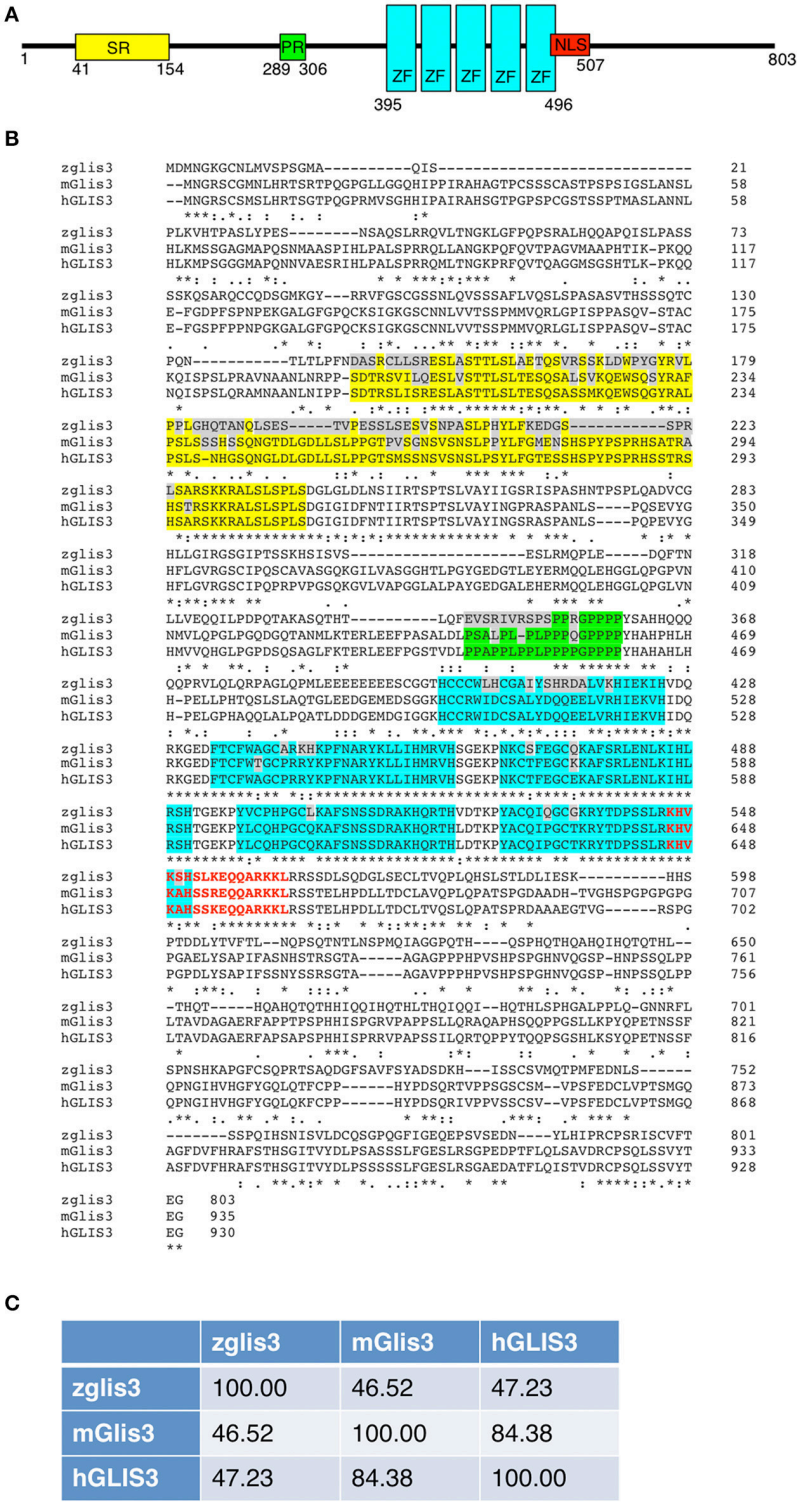
**(A)** Schematic representation of the zebrafish glis3 protein (UniProt F1QB13). Yellow, Serine-Rich (SR) domain; Green, Proline-Rich (PR) domain; Light Blue, Zinc-Finger (ZF) domain; Red, Nuclear Localization Signal (NLS). **(B)** Alignment of the *GLIS3* protein sequencing of the zebrafish (UniProt F1QB13), mouse (UniProt Q0GE24), and human (UniProt Q8NEA6). The color code of the different domains is reported above. The residues that differ between species are highlighted in gray. The missense *GLIS3* variants identified in NDH (orange) and CH (green) patients are reported. **(C)** Aminoacid identity between zebrafish, murine and human *GLIS3* proteins, performed using ClustalW2 software.

Regarding the missense variations identified in NDH or CH patients no functional studies are available so far. In Figure 1B we report the alignment of the aminoacid sequences of zebrafish, mouse and human *GLIS3*. The residues C536, H561, and R589 associated to NDH pathology localized within the zinc-finger domain and are completely conserved across species. Concerning the CH patients (12, 13), only 2/11 missense variants, affect aminoacids (D512 and E515) belonging the first zinc-finger, but are not conserved between human and zebrafish.

In addition, a number of genome wide association studies (GWAS) have associated several *GLIS3* single nucleotide polymorphism (SNP) with elevated circulating TSH levels and low T4 (1).

Taken together, all of these data support a relevant role of *GLIS3* in thyroid function and in the onset of CH, possibly contributing to the explanation of the missing hereditability of CH and the prevalent sporadic presentation of the disease (13).

Although hypothyroidism is associated with *GLIS3* variants, the absence of consistent pathological features in patients makes unclear the identification of a causative mechanism (8). High TSH and low T4 characterize all of twelve NDH patients reported so far, but in presence of a wide spectrum of structural thyroid abnormalities including athyreosis, hypoplasia, perifollicular, and interstitial fibrosis, and normal thyroid anatomy. Similar phenotypic variability was previously observed in patients with mutations in *NKX2.1* and *PAX8* genes, involved in the specification of thyroid primordium and in the subsequent expression of genes (*TG, TPO, TSHR*, and *NIS*) responsible for the differentiation and proliferation of thyroid follicles and the synthesis of thyroid hormones (TH) (14, 15). One possible explanation for the variable CH manifestations could be attributed to the tissue-specific expression of *GLIS3* transcripts of variable length. Large deletions or frameshift mutations causing profound disruptions of Glis3 structure are associated with the most severe manifestations of NDH. Instead, milder phenotypes appear correlated to missense substitutions that preserve a residual function of one or more *GLIS3* transcripts (8, 15).

An additional challenge of *GLIS3* defect is the high variability in the sensitivity of TSH suppression during thyroxine replacement and the consequent difficult TSH normalization (8). Several patients with elevated TSH and TG levels appear to be resistant to increasing doses of thyroxine therapy despite normalization of free T4, thus excluding alterations in the L-T4 absorption (4, 15).

Therefore, additional work is required to understand the genotype-phenotype correlations and to determine whether Gils3 acts upstream of genes involved in pathways regulating thyroid development, hormonogenesis, and the peripheral response to T4.

## *Glis3* murine models

Up to now, the murine model has been extensively used to investigate the pathological mechanism underlying the multisystem clinical presentation of NDH syndrome, and most of the researches have been focusing on pancreas development and the onset of diabetes.

The *Glis3*-null mice die within 1 week after birth due to the severity of neonatal diabetes (11). In mutant mice, neonatal diabetes is associated with decreased insulin levels caused by an impaired β-cell mass (11, 16), suggesting a relevant role for *Glis3* in the regulation of β-cell development and endocrine function (17).

In fact, *Glis3* overlaps with *Pdx1* and *Nkx6.1* expression in endocrine progenitors, and becomes mainly restricted to *Ins*+ β-cell in later stages (18). ZeRuth and co-workers demonstrated that Glis3 binds the insulin (*Ins*) promoter enhancing its transcription. Glis3 recruits Cbp/p300, which constitutes a scaffold for the formation of a larger transcriptional regulatory complex, including the other synergic factors Pdx1, Neurod1, and Mafa at the level of the insulin promoter (17). All of these studies indicated that *GLIS3* has multiple critical regulatory functions during pancreas development and also in the adult pancreas: regulating the development of endocrine progenitors, the generations and maturation of pancreatic β-cells, regulation of insulin expression as well as in maintaining normal duct morphology (8).

As for NDH patients, the *Glis3* mutant mice also developed polycystic kidney disease (19), pointing up the role of *Glis3* in the maintenance of the normal renal function. Kang and co-workers demonstrated that *Glis3* and its co-activator Wwtr1/TAZ are part of transcriptional regulatory networks that are critical in the function of the primary cilium (19).

As far as thyroid gland is concerned, the Refetoff and Jetten groups recently described that *Glis3* is essential for TH biosynthesis and postnatal thyroid gland development in mouse, acting down-stream of TSH/TSHR system (14). In mice, *GLIS3* appears to retain a relevant role in the expression of several functional proteins, such as the sodium/iodide symporter (Nis encoded by *SCL5A5*) and Pendrin (encoded by *SLC26A4*). In addition, despite a relevant role in thyroid cell proliferation, no significant thyroid developmental defects were observed in *Glis3* knock-out mice. These data are partially in contrast to those observed in the NDH patients, in which thyroid dysgenesis is a common feature. However, several factors, including the type and site of the mutation, can contribute the variable expression of the thyroid phenotype (14).

For this reason, we believe that the murine models available today do not fully recapitulate the clinical manifestations of the NDH patients. A similar condition was previously experienced with the knockout of *MCT8* gene in mice that fails to recapitulate the neurological manifestations of the human disease (20). This evidence made it clear that a single species would not suffice to obtain insights into the underlying pathological mechanisms. The evolutionary conservation of thyroid hormone action on neurodevelopment as well as the components regulating thyroid hormone signaling however offered the opportunity to answer different aspects of MCT8 function in brain development using different vertebrate species, such as zebrafish (21). In Mct8-deficient zebrafish embryos, it was possible to obtain the unprecedented evidence of an impaired thyroid hormone uptake at the level of the blood-brain barrier during the peri- and postnatal development (21).

Therefore, in this perspective we address the possibility of zebrafish as a viable vertebrate model to study in more details the role of *GLIS3* in thyroid morphogenesis and dysfunction.

## Zebrafish as a new model to study *glis3* functions

Zebrafish (*Danio rerio*) has emerged as an important and useful model system to study different human diseases. Zebrafish possesses a unique combination of features that makes it particularly well-suited for experimental and genetic analysis of vertebrate development. It has a short reproductive cycle with external fertilization and lays a large numbers of embryos per mating that can be analyzed under microscope because of their optical transparency. The availability of several genetic manipulation strategies, at costs that are definitely lower than in rodents, lead to the generation of zebrafish models for a wide variety of human diseases (22).

Like the other vertebrates, the basic unit of the zebrafish thyroid gland is the thyroid follicle, which arises from the pharyngeal endoderm (23). At 22 h post-fertilization (hpf) the zebrafish thyroid primordium starts to express the transcription factor *nkx2.4, pax2a, pax8, hhex*, and *foxe1*. As described in mice, knockdown experiments in zebrafish demonstrated that the transcription factor *nkx2.4, pax2a*, and *hhex* are required for the specification and the differentiation of thyroid primordium (24, 25). The differentiation of thyroid precursors continues up to 2 days post-fertilization (dpf) with the expression of the functional thyroid genes *tg, tpo*, and *slc5a5*. At 3 dpf the zebrafish thyroid follicles are completely developed and start to produce thyroid hormone (24).

Unlike mammals, the zebrafish thyroid is not organized as a compact gland, but is composed of single follicles localized along the ventral aorta (23). In fact, alterations in the architecture of the pharyngeal vessels are associated with severe thyroid defects, consistent with a correlation between thyroid morphology and vascular development (25). The proper amount of TH is tightly regulated by the hypothalamus-pituitary-thyroid axis, which is also preserved in zebrafish (24). As a consequence, this model has been extensively used to study the molecular mechanism underlying alteration in thyroid development (23, 25, 26) and function (27, 28). However, the mechanisms and the genes accounting for the commitment of the thyroid precursors from the endoderm are presently unknown.

The *glis3* gene is evolutionary conserved across species dating back to fishes (2) and in zebrafish it is localized on the chromosome 10 and encodes a protein of 804 residues that is 88.45 kD in size. A second transcript that encodes protein of 787 residues still not characterized to date. Although the aminoacid identity between zebrafish and human is rather low (47.23%), the glis3 protein structure and the five zinc-finger motives are well-conserved also in zebrafish (Figure 1).

**Figure 2:**
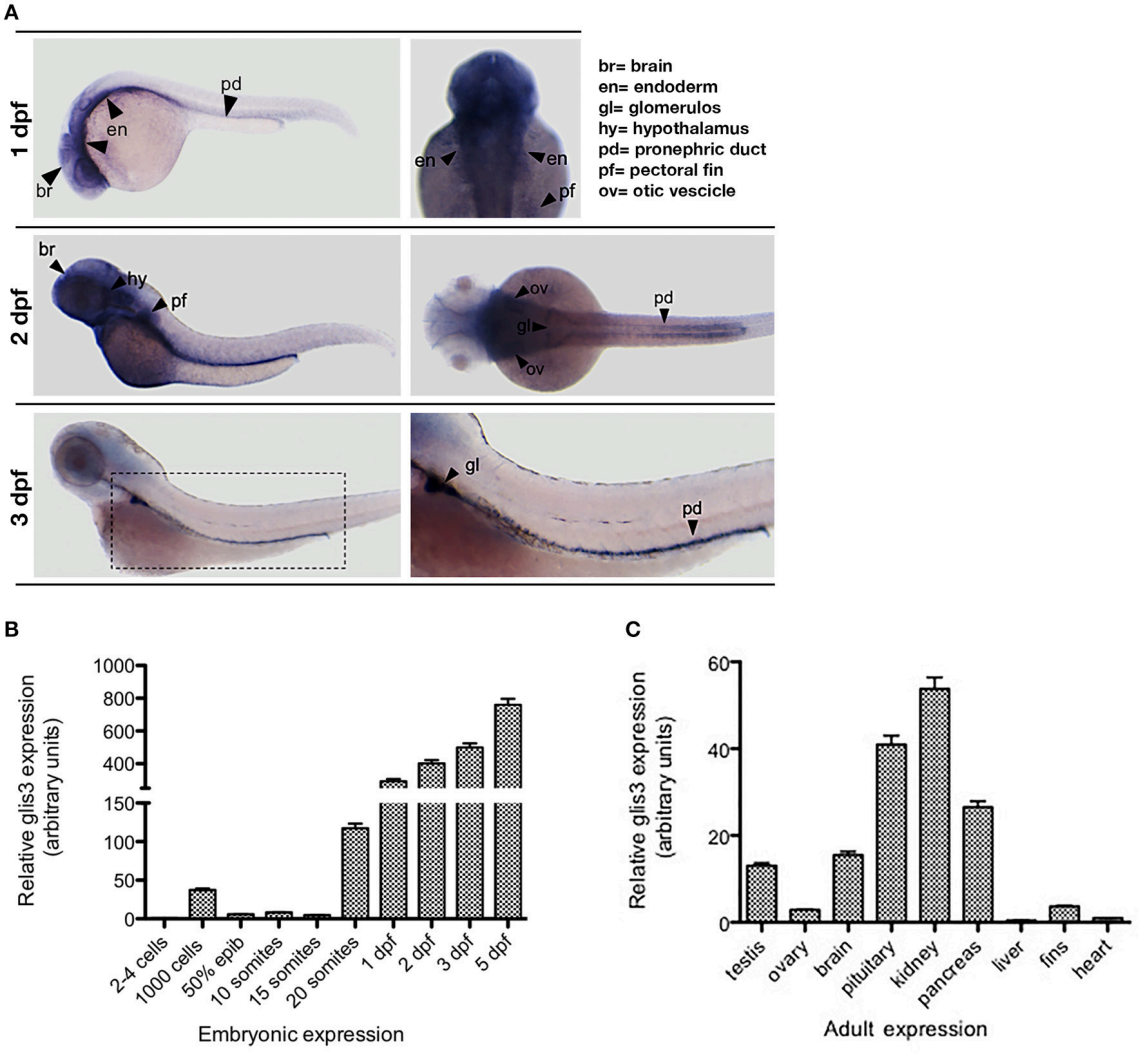
Ontogenetic pattern of *glis3* expression in zebrafish. **(A)** Tissue specific expression of *glis3*, at different developmental stages, analyzed by ISH using *glis3* antisense riboprobe followed by NBT/BCIP staining, as previously described (29). Images are representative of three experiments (30 embryos each). **(B,C)** qRT-PCR of *glis3* relative mRNA expression during embryonic development and adult tissues. Total RNA is extracted from pools of 50 embryos/stage and from 3 adults, respectively. cDNA synthesis and qRT-PCR following standard procedures (28). Experiments are performed in triplicate and results are expressed by Mean ± SD.

By *in situ* hybridization (ISH), we show that the zebrafish *glis3* transcript is expressed since the early developmental stages with an intense signal at the pharyngeal endoderm, brain and in the glomerulus and the pronephric ducts. Of note, *glis3* transcripts are not detectable when the thyroid gland development is completed at 3 dpf (Figure 2A). During embryonic development *glis3* starts to be expressed during somitogenesis with a peak at 20-somite stage (19 hpf). Afterwards, the *glis3* levels increase rapidly until the embryonic transition to the larval stage (Figure 2B). Consistent with clinical and experimental findings in humans and mice models, *glis3* is expressed in a tissue specific manner in the adult zebrafish. It is detectable at high level in kidney, pituitary and pancreas and at lower levels in testis, ovary, brain, and pectoral fins (Figure 2C).

The only available findings about the *glis3* function in zebrafish are now-a-days related to pancreas. The *glis3* knockdown embryos present Type 2 Diabetes (T2D) and a β-cell mass deficiency (30), confirming findings in mice (11).

Both *Glis3* and *Wwtr1* are known to contribute to renal function (19). Interestingly, *wwtr1* has been shown to have an important function in the antero-posterior pattering of the pronephric progenitor field (31) as well as in the differentiation of the thyroid gland (32) in zebrafish. It is therefore tempting to hypothesize a possible interaction between *glis3* and *wwtr1* during thyroid growth. To date, the involvement of *glis3* in the development and function of the thyroid gland and of the hypothalamic-pituitary-thyroid axis in zebrafish is presently unknown. The expression of *glis3* at the pharyngeal endoderm before the appearance of *nkx2.4* and *pax2a* expression in the thyroid primordium suggests a possible role for *glis3* during the specification of the thyroid precursors, and this hypothesis is currently under investigation in our Lab.

## Conclusion

To date, it is known that *GLIS3* is a pleiotropic master regulator of several pathways involved in the development and function of a broad range of tissues and organs. In particular, *GLIS3* is involved in the specification of several endocrine glands. Since the endocrine pancreas and thyroid arise from the same embryonic sheet, it is reasonable to think that *GLIS3* could act at both sites with similar mechanisms. However, based on current findings it is possible to envisage a dual role for *GLIS3* during the thyroid development and regulating thyroid function in the postnatal period.

The bewildering variability of the clinical manifestations of NDH and CH associated with *GLIS3* variants cannot be explained at present. Hence, there is need to improve our understanding of the pathophysiology underlying *GLIS3* action.

The establishment of a zebrafish model may constitute a valid alternative to mice in the definition of the early effects of *glis3* deficiency and, eventually, provide interesting insights on the molecular mechanisms involved in thyroid development and CH pathogenesis.

## Ethics statement

The protocol for zebrafish studies was approved by the Ethic Committee of Istituto Auxologico Italiano (study 05C102_2011).

## Author contributions

GR performed the zebrafish experiments and prepared the draft of the manuscript. FM supervised the zebrafish study and contributed the draft preparation. LP conceived the study, obtained research funds and finalized the manuscript. All authors approved the final version of the manuscript.

### Conflict of interest statement

The authors declare that the research was conducted in the absence of any commercial or financial relationships that could be construed as a potential conflict of interest.
